# Sleep quality, quality of life, fatigue, and mental health in COVID-19 post-pandemic Türkiye: a cross-sectional study

**DOI:** 10.3389/fpubh.2024.1250085

**Published:** 2024-02-23

**Authors:** Abdulbari Bener, Ebru Morgul, Mahmut Tokaç, Antonio Ventriglio, Timothy R. Jordan

**Affiliations:** ^1^Department of Biostatistics and Medical Informatics, School of Medicine, Istanbul Medipol University, Istanbul, Türkiye; ^2^Department of Evidence for Population Health Unit, School of Epidemiology and Health Sciences, The University of Manchester, Manchester, United Kingdom; ^3^Department of Psychology, School of Humanities and Social Sciences, Ibn Haldun University, Istanbul, Türkiye; ^4^Department of Medicine and Education Unit, School of Medicine, Istanbul Medipol University, Istanbul, Türkiye; ^5^Department of Clinical and Experimental Medicine, University of Foggia, Foggia, Italy

**Keywords:** COVID-19, sleep quality, quality of life, mental health, depression, anxiety, stress

## Abstract

**Aim:**

This study explores the predictors and associated risk factors of sleep quality, quality of life, fatigue, and mental health among the Turkish population during the COVID-19 post-pandemic period.

**Materials and methods:**

A cross-sectional survey using multi-stage, stratified random sampling was employed. In total, 3,200 persons were approached. Of these, 2,624 (82%) completed the questionnaire package consisting of socio-demographic information, Pittsburgh Sleep Quality Index (PSQI), the WHO Quality of Life Brief Version (WHOQOL-BREF), Fatigue Assessment Scale (FAS), Patients Health Questionnaire (PHQ-15), GAD-7 anxiety scale, and the 21-item Depression, Anxiety, Stress Scale (DASS-21).

**Results:**

Significant differences between genders were found regarding socio-demographic characteristics (*p* < 0.01). Using PHQ-15 for depressive disorders, significant differences were found between normal and high severity scores (≥ 10), regarding age group (*p* < 0.001), gender (*p* = 0.049), educational level (*p* < 0.001), occupational status (*p* = 0.019), cigarette smoking (*p* = 0.002), waterpipe-narghile smoking (*p* = 0.039), and co-morbidity (*p* = 0.003). The WHOQOL-BREF indicated strong correlations between public health, physical health, psychological status, social relationships, environmental conditions, and sleep disorders (*p* < 0.01). Furthermore, comparisons of the prevalence of mental health symptoms and sleeping with PHQ-15 scores ≥ 10 (*p* = 0.039), fatigue (*p* = 0.012), depression (*p* = 0.009), anxiety (*p* = 0.032), stress (*p* = 0.045), and GAD-7 (*p* < 0.001), were significantly higher among the mental health condition according to sleeping disorder status. Multiple regression analysis revealed that DASS21 stress (*p* < 0.001), DASS21 depression (*p* < 0.001), DASS21 anxiety (*p* = 0.002), physical health (WHOQOL-BREF) (*p* = 0.007), patient health depression-PHQ-15 (*p* = 0.011), psychological health (WHOQOL-BREF) (*p* = 0.012), fatigue (*p* = 0.017), and environmental factors (WHOQOL-BREF) (*p* = 0.041) were the main predictor risk factors associated with sleep when adjusted for gender and age.

**Conclusion:**

The current study has shown that sleep quality was associated with the mental health symptoms of depression, anxiety, stress, and fatigue. In addition, insufficient sleep duration and unsatisfactory sleep quality seemed to affect physical and mental health functioning.

## Introduction

According to the 2023 report by the WHO, the COVID-19 pandemic, which started in November 2019, produced over 760 million confirmed cases worldwide, and over 6.9 million reported deaths as of August 2023. Since the beginning of the pandemic, significant measures have been taken across the world, such as travel restrictions and home confinements, to control the pandemic. With the progress achieved in the management of the pandemic through the year 2021, the restrictions were gradually lifted. However, more than 3 years since the onset of the COVID-19 pandemic, serious impacts on many aspects of public health are still being observed, including sleep disturbances and mental health symptoms, along with various physiological symptoms (1, 2). Therefore, it is important to explore how the pandemic situation affected public health in order to reduce negative consequences and promote changes in lifestyles and improvements in overall health and life quality among the population. However, pandemic conditions have left an ongoing impact on the physical and mental health of many individuals regardless of whether they had the infection or not. The current research focuses particularly on sleep quality, quality of life, and several mental health symptoms during the aftermath of pandemic restrictions among the Turkish population.

Sleep disturbances, such as difficulty falling asleep or maintaining sleep, are quite common in many countries around the world (3, 4). Since poor sleep quality is linked to many medical and psychological disorders including obesity, cardiovascular diseases, and depression, and has a direct effect on life quality, investigating these effects of sleep quality is important (5–8). Several neurocognitive dysfunctions such as attention deficits and cognitive performance impairment, as well as psychological disorders such as stress, depression, anxiety, and impulse control problems, are related to sleep disturbances (5, 9). If untreated, such sleep-related deficits may lead to further potentially health-threatening consequences such as increased risk of cardiovascular diseases (4). Furthermore, disturbed sleep quality also has important consequences on quality of life, as it can lead to daytime impairments that affect people's performance at work and quality of social life (4, 5).

Since the outbreak of the COVID-19 pandemic, multiple studies have shown increased sleep disturbances due to factors related to the pandemic [8, 1, 9, (10)]. A systematic review and meta-analysis investigating changes in the quality of sleep and disturbances of sleep among the general population prior to and during the COVID-19 lockdown has revealed a decline in sleep quality and sleep efficiency, and an increase in sleep disturbances (11). The worldwide pandemic created an unpredictable, stressful, anxiety-provoking environment, with fear of infection and financial concerns added to social isolation and other negative consequences of lockdowns (12–14). Home confinement also resulted in increased screen time as many individuals turned to the media and the internet for information on the pandemic or to distract themselves from the stressful situation. These psychological and physical conditions may have had a negative impact on the quality of sleep experienced by the general population (13, 15–17).

In the initial phases of the pandemic, people with mental health problems in the general population were reported to be struggling with higher levels of depression, anxiety, stress, and fear symptoms (6, 12, 18–24). Indeed, several studies conducted in various countries have reported the negative effects of the COVID-19 pandemic on the mental health of the general population (1, 14). Multiple studies have also shown associations among depression, anxiety, stress, and negative changes in sleep (13, 15, 25, 26).

Although the number of infection cases has dramatically declined and most of the world has lifted restrictions concerning COVID-19, it is still important to assess the long-term health consequences of experiencing the pandemic among the general population.

Accordingly, the objective of this study is to examine the predictors and associated risk factors of sleep quality, quality of life, fatigue, and mental health among the Turkish population during the post-COVID period. For this purpose, several demographic variables, habits, and living conditions were explored as predictor variables of the outcome health variables. In addition, based on the literature reviewed, poor sleep quality is expected to be correlated with negative mental health symptoms, fatigue, and poor quality of life.

## Participants and methods

The research used a cross-sectional multi-center-based survey, conducted among the urban and rural populations of Istanbul, including men and women (aged 20 years and over). The sample size calculations were derived from the following parameters: a margin of error of 2.0% with a confidence level of 99%, and an estimated sample proportion of 25% to be considered. Accordingly, a multi-stage total of 3,200 individuals were approached. A total of 2,624 (82%) participants completed the questionnaire between 1 January and 31 December 2022.

### Measurements

#### World health organization quality-of-life assessment

The WHOQOL-BREF (27) has 26 questions consisting of four domains: physical health, psychological status, social relationships, and environmental conditions. The WHOQOL-BREF has been shown to have good reliability and validity in a number of different populations.

#### Patients heath questionnaire - depression

The PHQ-15 is a screening tool for depressive disorders (28). The validity and reliability of the Turkish version of the PHQ-15 exhibited satisfactory internal consistency (Cronbach's alpha = 0.78). Total scores can range from 0 to 30, resulting in the following categories: 0–4 none, 5–9 mild, 10–14 moderate, and 15–30 severe. The recognized cut-off value is 10.

#### Depression anxiety stress scale

The 21-item DASS-21 by Lovibond and Lovibond (24) was used to assess depression, anxiety, and stress. Cronbach's alpha internal consistency coefficient was found as α = 0.86 for the depression subscale, α = 0.84 for the anxiety subscale, and α = 0.80 for the stress subscale in the current study. The scale categorizes the participants into five categories: normal, mild, moderate, severe, and very severe.

#### Generalized anxiety disorder screener

The GAD-7 is a simple instrument containing seven items that track anxiety symptoms (29). The reliability coefficient of Cronbach's α for the overall GAD-7 scale is 0.86, which is greater than the recommended value of 0.80, suggesting excellent reliability. The total scores range from 0 to 21 and were categorized as follows: minimal/no anxiety (0–4), mild (7–9, 13, 15), moderate (11, 12, 14, 16, 17), and severe (1, 18–23).

#### Pittsburgh sleep quality index

The Pittsburgh Sleep Quality Index (PSQI) was developed by Buysse et al. (30). The reliability and validity of PSQI in the current study population was 0.84. The categorization of the total scores of PSQI is as follows: PSQI ≤ 5= “Good sleep quality,” PSQI 6–8= “Average sleep,” and PSQI ≥ 9 = “Poor sleep.”

#### Fatigue assessment scale

The Fatigue Assessment Scale (FAS), developed by Michielsen et al. (31), is a 10-item self-report questionnaire intended to assess general fatigue. The FAS displayed good internal consistency (Cronbach's alpha = 0.91). A total FAS score ranging from 10 to 21 indicates no fatigue (normal) and FAS scores ranging from 22 to 50 indicate fatigue.

### Statistical analyses

SPSS v25 was utilized to analyze the present data, and percentages were computed for each categorical variable. To assess the normal distribution of the data, the Kolmogorov-Smirnov test and normality plots were utilized. Student's *t*-test was used to ascertain the significance of differences between mean values. The chi-square test was used to determine significant differences between categorical variables. A multivariate stepwise regression analysis was performed to predict potential risk factors (determinants) for sleep and mental health. Furthermore, the multiple stepwise regression analysis was used to control for the effects of gender and age. All statistical tests were two-tailed, and *p* < 0.01 was considered significant.

## Results

A significant difference was found between men and women in educational level, occupational status, monthly income, place of residence as urban or rural, number of rooms, and number of family members (*p* < 0.01) (Table 1). The educational status ranged from primary school to university degree and postgraduate levels. In terms of occupational status, the majority were in professional/sedentary and clerical occupation categories.

**Table 1:** Socio-demographic characteristics of participants by gender (*N* = 2,624).

		**Men= 1,420**	**Women= 1,204**	***p*-value significance**
		***n*** **(%)**	***n*** **(%)**	
**Age group**	< 30	425 (29.9)	338 (28.1)	
	30–39	312 (22.0)	274 (22.8)	
	40–49	234 (16.5)	264 (21.9)	0.004
	50–59	263 (18.5)	188 (15.6)	
	= >60	186 (13.1)	140 (11.6)	
**Educational level**				
	Preparatory	115 (8.1)	12 (1.0)	
	Secondary	247 (17.4)	88 (7.4)	
	High school	465 (32.7)	101 (8.4)	0.001
	University / College	420 (29.6)	819 (68.0)	
	Postgraduate M.Sc. / PhD	173 (12.2)	183 (15.2)	
**Occupation status**				
	Professional/ Sedentary	386 (27.2)	434 (36.0)	
	Administrative/ Clerical	476 (33.5)	344 (28.6)	
	Manual labor	218 (15.4)	31 (2.6)	0.001
	Housewife	0	312 (25.9)	
	Business	340 (23.9)	83 (6.9)	
**Income**	Low	534 (46.0)	381 (31.6)	
	Medium	415 (30.3)	413 (34.0)	0.001
	High	471 (23.7)	410 (34.4)	
**Physical exercise**				
	Yes	391 (27.5)	322 (26.7)	0.627
	No	1,029 (72.5)	234 (73.3)	
**Number of rooms**				
	= < 3 rooms	783 (55.1)	646 (53.7)	0.103
	> 3 rooms	637 (44.9)	558 (46.3)	
**Number of family members**				
	= < 5 people	650 (45.8)	593 (49.3)	0.049
	> 5 people	770 (54.2)	611 (50.7)	
**Cigarette smoker**				
	Yes	357 (25.1)	239 (19.9)	0.001
	No	1,063 (74.9)	965 (80.1)	
**Nargile-waterpipe smoker**				
	Yes	278 (19.6)	174 (14.5)	0.001
	No	1,142 (80.4)	1,030 (85.5)	
**Co-morbidity**				
	None	1,041 (73.3)	998 (82.9)	
	One	308 (21.7)	140 (11.6)	0.004
	Two	71 (5.0)	66 (5.5)	

*n* = *the number of participants within each subgroup*.

Table 2 provides participants' characteristics, lifestyle behavior, and level of depression using PHQ-15, and the results indicate significant differences between normal and high severity regarding age group (*p* < 0.001), gender (*p* = 0.049), educational level (*p* < 0.001), occupational status (*p* = 0.019), smoking cigarettes (*p* = 0.002), waterpipe-narghile smoking (*p* = 0.039), and co-morbidity (*p* = 0.003). Smoking cigarettes was reported by 25% of the participants, and narghile waterpipe use was reported by 20%.

**Table 2:** Participants' characteristics, lifestyle behavior, and depression using PHQ-15 tools (*N* = 2,624).

		***N* = 1,751; PHQ-15 < 10 none-mild**	***N* = 873; PHQ−15 ≥10** **moderate-severe**	***p*-value significance**
		***n*** **(%)**	***n*** **(%)**	
**Age group**	< 30	585 (33.4)	178 (20.4)	
	30–39	349 (19.9)	237 (27.1)	
	40–49	334 (19.1)	164 (18.8)	0.001
	50–59	306 (17.5)	145 (16.6)	
	= >60	177 (10.1)	149 (17.1)	
**Gender**				
	Men	924 (52.8)	496 (56.8)	0.049
	Women	827 (47.2)	377 (43.2)	
**ducational level**				
	Preparatory	81 (4.6)	46 (5.3)	
	Secondary	223 (12.7)	113 (12.9)	
	High school / College	333 (19.0)	233 (26.7)	0.001
	University	868 (49.6)	371 (42.5)	
	Postgraduate M.Sc. / PhD	246 (14.8)	110 (12.6)	
**Occupational status**				
	Professional / Sedentary	559 (31.9)	261 (29.9)	
	Administrative / Clerical	537 (30.7)	283 (32.4)	
	Manual Labor	177 (10.1)	72 (8.2)	0.019
	Housewife	220 (12.6)	92 (10.5)	
	Business	258 (14.7)	165 (18.9)	
**Physical activity**				
	Yes	481 (27.5)	232 (26.6)	0.627
	No	1,270 (72.5)	641 (73.4)	
**Number of rooms**				
	= < 3 rooms	934 (53.3)	495 (56.7)	0.103
	> 3 rooms	817 (46.7)	378 (43.3)	
**Number of family members**				
	= < 5 people	809 (46.2)	434 (49.7)	0.049
	> 5 people	942 (53.8)	439 (50.3)	
**Cigarette smoker**				
	Yes	404 (23.1)	155 (17.8)	0.002
	No	1,347 (76.9)	718 (82.2)	
**Nargile-waterpipe smoker**				
	Yes	349 (19.6)	142 (16.3)	0.039
	No	1,408 (80.4)	731 (83.7)	
**Co-morbidity**				
	None	1,340 (76.6)	699 (80.1)	
	One	328 (18.7)	120 (13.7)	0.003
	Two	831 (4.7)	54 (6.2)	

Table 3 shows the prevalence of mental health symptoms by gender. The prevalence of depression was 30% among women and 20% among men. The prevalence of mental health symptoms is significantly higher among women than men regarding depression (*p* < 0.001), anxiety (*p* < 0.001), stress (*p* = 0.031), GAD-7 (*p* = 0.030), and PSQI (*p* = 0.010).

**Table 3:** Prevalence of mental health symptoms by gender (*N* = 2,624).

**Variables**	**Men = 1,420 Yes *n* (%)**	**Women = 1,204 Yes *n* (%)**	***p*-value significance**
**GHQ-15**			
None	528 (37.2)	489 (40.6)	
Mild	396 (27.9)	338 (28.1)	
Moderate	297 (20.9)	219 (18.2)	0.185
Severe	199 (14.0)	158 (13.1)	
**Fatigue**			
Normal	911 (64.2)	783 (63.4)	0.678
Fatigue	509 (35.8)	441 (38.6)	
**Pittsburgh sleep quality index**			
Good sleep	507 (35.7)	385 (32.0)	
Average sleep	447 (31.5)	356 (29.6)	0.010
Poor sleep	466 (32.8)	463 (38.5)	
**DASS21 depression**			
Normal	442 (31.1)	309 (25.7)	
Mild	321 (22.6)	297 (24.7)	
Moderate	298 (21.0)	237 (19.7)	0.001
Severe	243 (17.1)	209 (17.4)	
Very severe	116 (8.2)	152 (12.6)	
**DASS21 anxiety**			
Normal	528 (37.2)	351 (29.2)	
Mild	430 (30.3)	441 (36.6)	
Moderate	170 (12.0)	177 (14.7)	0.001
Severe	161 (11.3)	117 (9.7)	
Very severe	131 (9.2)	118 (9.8)	
**DASS21 stress**			
Normal	504 (35.5)	404 (33.6)	
Mild	297 (20.9)	244 (20.3)	
Moderate	262 (18.5)	210 (17.4)	0.031
Severe	213 (15.0)	174 (14.5)	
Very severe	1,443 (10.1)	172 (14.3)	
**GAD-7 anxiety**			
Minimal	510 (35.9)	417 (34.6)	
Mild	371 (26.1)	267 (22.2)	0.010
Moderate	375 (26.4)	360 (29.9)	
Severe	164 (14.5)	160 (13.3)	

Table 4 presents the quality of life descriptive with the test for normality of distribution and reliability of the domains. The WHOQOL-BREF domains were all normally distributed. Furthermore, Table 5 reveals higher statistically significant positive correlations between WHOQOL-BREF public health, physical health, psychological status, social relationships, environmental conditions, and sleeping disorders (*p* < 0.01). The correlation between WHOQOL-BREF physical health and sleeping disorders was *r* = 0.40, *p* < 0.01.

**Table 4:** Quality of life descriptive statistics with the test for normality of distribution and reliability of the domains.

**Domain**	**Mean**	**Median**	**Standard deviation**	**Minimum**	**Maximum**	***P-*value**	**Cronbach alpha**
Physical health	13.46	14.00	2.75	4.85	19.72	< 0.001	0.797
Psychological health	13.95	14.00	2.91	4.34	20.00	< 0.001	0.840
Social relationships	14.87	14.67	3.16	4.06	20.00	< 0.001	0.818
Environment	15.28	15.00	3.04	5.18	21.54	< 0.001	0.805
Rate your quality of life	3.99	4.00	0.93	1.00	5.00	< 0.001	0.826
How satisfied are you with your health?	3.77	4.00	0.87	1.00	5.00	< 0.001	0.826

**Table 5:** Pearson's Correlation between sleeping disturbances and mental health parameters (*N* = 2,624).

**Variables**	**Sleeping disturbances correlations coefficient (r)**	*P*-value ^*^significance
GHQ-15 general health questionnaire	*r* = 0.335	0.001
Fear	*r =* 0.594	0.001
Fatigue	*r =* 0.486	0.001
DASS21 depression symptoms	*r =* 0.304	0.001
DASS21 anxiety symptoms	*r =* 0.178	0.050
DASS21 stress symptoms	*r =* 0.164	0.001
GAD-7 anxiety	*r =* 0.311	0.001
**A WHOQOL-BREF quality of life assessment:**		
Public health	*r =* 0.260	0.005
Physical health (WHOQOL-BREF)	*r =* 0.273	0.005
Psychological health (WHOQOL-BREF)	*r =* 0.829	0.001
Social relationships	*r =* 0.246	0.005
Environmental factors (WHOQOL-BREF)	*r =* 0.494	0.001

Table 6 shows the prevalence of mental health symptoms and sleeping disorders. The prevalence of PHQ-15 scores ≥ 10 was 10% among people with sleeping disorders and 5% among people without sleeping disorders. The prevalence of PHQ-15 scores ≥ 10 was 10% among people with sleeping disorders and 5% among people without sleeping disorders. As can be seen, the prevalence of PHQ-15 ≥ 10 scores (*p* = 0.039), fatigue (*p* = 0.012), depression (*p* = 0.009), anxiety (*p* = 0.032), stress (*p* = 0.045), and GAD-7 (*p* < 0.001) were significantly higher among the mental health condition compared to sleeping disorder status.

**Table 6:** Prevalence of mental health symptoms and sleeping disorders (*N* = 2,624).

**Variables and scores**	**Sleep quality**			***p*-value**
	**Good sleep** = **892 (PSQI score**<**5) Yes** ***n*** **(%)**	**Average sleep** = **830 (PSQI score 6–8) Yes** ***n*** **(%)**	**Poor sleep** = **902 (PSQI score** > **8) Yes** ***n*** **(%)**	**Significance**
**GHQ-15**				
None	372 (41.7)	290 (34.9)	355 (39.4)	
Mild	235 (26.3)	254 (30.6)	245 (27.2)	0.026
Moderate	183 (20.5)	167 (20.1)	166 (18.4)	
Severe	102 (11.4)	119 (14.3)	136 (151)	
**Fatigue**				
Normal	549 (61.5)	504 (62.0)	621 (67.6)	0.012
Fatigue	343 (68.5)	309 (38.0)	298 (32.4)	
**DASS21 depression**				
Normal	268 (30.0)	229 (27.6)	254 (28.2)	
Mild	222 (24.9)	198 (23.9)	198 (24.1)	
Moderate	200 (24.4)	151 (18.2)	184 (22.0)	0.009
Severe	121 (13.6)	159 (19.2)	172 (19.1)	
Very severe	81 (9.1)	93 (102)	94 (10.4	
**DASS21 anxiety**				
Normal	314 (35.2)	288 (35.9)	277 (30.5)	
Mild	285 (32.0)	261 (32.5)	325 (35.0)	
Moderate	107 (12.0)	114(14.2)	126 (13.6)	0.032
Severe	101 (11.3)	81 (10.1)	96 (10.3)	
Very severe	85 (9.5)	59 (7.3)	105 (11.3)	
**DASS21 stress**				
Normal	292 (32.7)	282 (34.0)	334 (37.0)	
Mild	174 (19.5)	181 (21.8)	186 (20.6)	
Moderate	170 (19.1)	141 (17.0)	161 (17.8)	0.045
Severe	153 (17.2)	130 (15.7)	104 (11.5)	
Very severe	103 (115)	96 (11.6)	117 (13.0)	
**GAD-7 anxiety**				
Minimal	331 (371)	271 (32.7)	325 (36.0)	
Mild	238 (26.7)	174 (21.0)	239 (265)	0.001
Moderate	227 (25.4)	259 (31.2)	236 (26.2)	
Severe	96 (10.8)	126 (13.2)	102 (11.3)	

Table 7 shows the relationships between sleeping disorders and mental health using multivariate stepwise regression analysis. It can be seen from this table that DASS21 stress (*p* < 0.001), DASS21 depression (*p* < 0.001), DASS21 anxiety (*p* = 0.002), physical health (WHOQOL-BREF) (*p* = 0.007), patient health depression-PHQ-15 (*p* = 0.011), psychological health (WHOQOL-BREF) (*p* = 0.012), fatigue (*p* = 0.017), and environmental factors (WHOQOL-BREF) (*p* = 0.041) were considered as the main predictor risk factors associated with sleep quality after adjusting for age and gender.

**Table 7:** The relationships between sleeping disorders and mental health using multivariate stepwise regression analysis (*N* = 2,624).

**Variables**	**Regression coefficient**	**Standard error**	***t*-test value**	**p-value significance**
DASS-21 stress symptoms	0.226	0.069	3.255	0.001
DASS-21 depression symptoms	0.980	0.221	4.518	0.001
DASS-21 anxiety symptoms	0.950	0.289	3.328	0.002
Physical health (WHOQOL-BREF)	0.620	0.230	2.709	0.007
Patient Health depression-PHQ-15	0.267	0.105	2.541	0.011
Psychological health (WHOQOL-BREF)	0.579	0.218	2.643	0.012
Fatigue	0.900	0.380	2.389	0.017
Environmental factors (WHOQOL-BREF)	0.169	0.083	2.043	0.041

*Model adjusted for age, gender; MS, Metabolic Syndrome (1* = *yes, 0* = *no)*.

## Discussion

Since the outbreak of COVID-19, the management and prevention of the negative health consequences of the pandemic have become a major concern. Globally, a significant emphasis has been placed on addressing and preventing the adverse effects of the COVID-19 pandemic on health since its onset, and this has emerged as a significant public health priority. Further research into the ongoing effects of COVID-19 is still needed to provide better insight into COVID-19-related physical and mental symptoms, and for the development of preventive measures and programs to promote healthy living (12, 18, 19, 23, 24). The findings of the current study contribute to this process by showing that negative health consequences of COVID-19 are still present in major populations.

The current research focused particularly on sleep quality, quality of life, fatigue, and mental health during the post-COVID-19 period. The findings indicated the prevalence of mental health symptoms and sleeping disorders among the Turkish population during the aftermath period of the pandemic. In addition, depression, stress, anxiety, physical and psychological health, fatigue, and environmental factors are shown as the main predictors of sleep quality in the current findings. The current findings concur with previous multinational research showing that people who experience increased symptoms of depression were more vulnerable to experiencing psychological burdens because of the COVID-19 pandemic (32). Furthermore, studies have reported increased depression, anxiety, and stress, and poor sleep quality (16, 33, 34), as well as increased mental and physical health conditions related to COVID-19 exposure among Chinese populations (13, 17).

COVID-19 has created considerable amounts of fear, fatigue, depression, anxiety, and stress in many communities, and is considered the greatest pandemic in centuries (12–14, 17, 23, 24, 32). Recently, studies conducted on populations in Istanbul, Turkey, found a high level of fatigue, stress, and fear associated with COVID-19 (12, 14, 24, 25). More recently, a systematic review and meta-analysis (11) comparing the effects of COVID-19 before and during lockdown, has shown a significant negative impact of the lockdown period in terms of changes in sleep quality among the general population. Consistently, the present study indicates the prevalence of poor sleep quality among Turkish populations and provides supporting evidence for the link between mental health symptoms and sleep disturbances. Studies examining associations between sleep quality and mental health symptoms often discuss the unclear direction of causality (3, 16). While mental health problems can result in the deterioration of sleep, on the other hand, sleep deprivation may lead to hormonal changes that can contribute to the development of mental health problems such as depression and anxiety. The present study suggests that the prevalence of sleep disorders remains high in the aftermath of the pandemic, and taking measures to improve sleep quality among the population should be considered a public health priority. Considering the main predictors and risk factors addressed in this research, implementing interventions to improve sleep quality among the general population could yield positive effects on both physical and mental health.

We are aware that our study has some limitations. First, we utilized a cross-sectional design, which may limit the ability to detect causal relationships. Second, the study was limited by the instruments employed to assess the variables. Our findings were derived from self-report assessments, and the assessment of mental health states did not involve clinical evaluation. The potential for recall bias and underreporting should therefore be considered. Third, since the study was based on self-administrated surveys, there is a possibility of selection bias in including participants based on their availability. On the other hand, one of the considerable strengths of the current study is its inclusion of a large sample size, enabling a thorough investigation that contributes valuable evidence supporting the link between sleep quality and mental health symptoms in a post-COVID-19 population.

## Conclusion

The current study revealed the prevalence of mental health symptoms (such as depression, stress, anxiety, and fatigue) among the general Turkish population who experienced the social trauma of the COVID-19 pandemic. The study provided supporting evidence concerning the association between sleep quality and mental health symptoms and indicated the substantial impact of the COVID-19 pandemic on this association. The findings show that, while negative mental health significantly contributes to poor sleep quality, insufficient sleep duration and unsatisfactory sleep quality have a negative impact on physical and mental health functioning. The current findings highlight the importance of providing adequate treatment and prevention strategies for sleep disorders as a public health concern, and this may help reduce the likelihood of the worsening, or onset, of several mental health disorders among the population.

## Data availability statement

The raw data supporting the conclusions of this article will be made available by the authors, without undue reservation.

## Ethics statement

The studies involving humans were approved by the Clinical Research Ethics Committee of Istanbul Medipol University, Institutional Review Board (Research Protocol IRB# E.10840098-604.01.01-0.14180 and IRB# E. 10840098-604.01.01- 1328). The studies were conducted in accordance with the local legislation and institutional requirements. The participants provided their written informed consent to participate in this study.

## Author contributions

AB, EM, and MT contributed to the conception, design, data collection, organization, statistical analysis, wrote the first draft of the article, they also contributed to the interpretation of the data, and writing and critical revision of the approved the final version of the manuscript. AV and TJ contributed to the conception, wrote the first draft of the article, contributed to the interpretation of the data and writing, and undertook critical revisions of the approved final version of the manuscript. All authors approved the final version.

## References

[1] Dos Santos Alves Maria G de Oliveira Serpa AL Ferreira CDMC de Andrade VD Ferreira ARH de Souza Costa D. Impacts of mental health in the sleep pattern of healthcare professionals during the COVID-19 pandemic in Brazil. J Affect Disord. (2023) 323:472–81. 10.1016/j.jad.2022.11.082 36455718 PMC9705011

[2] Taheri M Irandoust K Reynoso-Sánchez LF Muñoz-Helú H Cruz-Morales KN Torres-Ramírez R. Effects of home confinement on physical activity, nutrition, and sleep quality during the COVID-19 outbreak in amateur and elite athletes. Front Nutr. (2023) 10:1143340. 10.3389/fnut.2023.1143340 37139442 PMC10150803

[3] de Souza Lopes C Robaina JR Rotenberg L. Epidemiology of Insomnia: Prevalence and Risk Factors. In:Sahoo S, editor. In Can't Sleep? Issues of Being an Insomniac Shanghai: In Tech (2012), 1–21.

[4] Mollayeva T Thurairajah P Burton K Mollayeva S Shapiro CM Colantonio A. The pittsburgh sleep quality index as a screening tool for sleep dysfunction in clinical and non-clinical samples: a systematic review and meta-analysis. Sleep Med Rev. (2016) 25:52–73. 10.1016/j.smrv.2015.01.009 26163057

[5] Manzar MD BaHammam AS Hameed UA Spence DW Pandi-Perumal SR Moscovitch A. Dimensionality of the pittsburgh sleep quality index: a systematic review. Health Qual Life Outcomes. (2018) 16:1–22. 10.1186/s12955-018-0915-x 29743066 PMC5944037

[6] Franceschini C Musetti A Zenesini C Palagini L Scarpelli S Quattropani MC. Poor sleep quality and its consequences on mental health during the COVID-19 lockdown in Italy. Front Psychol. (2020) 11:3072. 10.3389/fpsyg.2020.574475 33304294 PMC7693628

[7] Taheri M Irandoust K. The exercise-induced weight loss improves self-reported quality of sleep in obese elderly women with sleep disorders. Sleep Hypn. (2018) 20:54–9. 10.5350/Sleep.Hypn.2017.19.0134

[8] Taheri M Irandoust K. The relationship between sleep quality and lifestyle of the elderly. Iran J Ageing. (2020) 15:188–99. 10.32598/sija.13.10.110

[9] Bener A Griffiths MD Cahit Barisik C Inan FC Morg E. Impacts of COVID-19 and lockdown on mental health: depression, anxiety, stress and fear among adult population in Turkey. Arch Clin Biomed Res. (2022) 6:1010–20. 10.26502/acbr.50170312ee

[10] Vindegaard N Benros ME. COVID-19 pandemic and mental health consequences: Systematic review of the current evidence. Brain Behav Immun. (2020) 89:531–42. 10.1016/j.bbi.2020.05.048 32485289 PMC7260522

[11] Limongi F Siviero P Trevisan C Noale M Catalani F Ceolin C. Changes in sleep quality and sleep disturbances in the general population from before to during the COVID-19 lockdown: a systematic review and meta-analysis. Front Psychiatry. (2023) 14:1166815. 10.3389/fpsyt.2023.1166815 37124253 PMC10134452

[12] Morgul E Bener A Atak M Akyel S Aktaş S Bhugra D. COVID-19 pandemic and psychological fatigue in Turkey. Int J Soc Psychiatry. (2021) 10:20764020941889. 10.1177/0020764020941889 32650681 PMC7355205

[13] Huang Y Zhao N. Generalized anxiety disorder, depressive symptoms and sleep quality during COVID-19 outbreak in China: a web-based cross-sectional survey. Psychiatry Res. (2020) 288:112954. 10.1016/j.psychres.2020.112954 32325383 PMC7152913

[14] Uysal B Görmez V Eren S Morgül E Öcal NB Karatepe HT. Living with COVID-19: Depression, anxiety and life satisfaction during the new normal in Turkey. J Cogn Psychother. (2021) 10:257. 10.5455/JCBPR.62468

[15] Salfi F Lauriola M D'Atri A Amicucci G Viselli L Tempesta D. Demographic, psychological, chronobiological, and work-related predictors of sleep disturbances during the COVID-19 lockdown in Italy. Sci Rep. (2021) 11:11416. 10.1038/s41598-021-90993-y 34075173 PMC8169862

[16] Gupta R Grover S Basu A Krishnan V Tripathi A Subramanyam A. Changes in sleep pattern and sleep quality during COVID-19 lockdown. Indian J Psychiatry. (2020) 62:370. 10.4103/psychiatry.IndianJPsychiatry_523_20 33165382 PMC7597722

[17] Sun QM Qin QS Chen BX Shao RF Zhang JS Li Y. Stress, anxiety, depression and insomnia in adults outside Hubei province during the COVID-19 pandemic. Zhonghua Yi Xue za Zhi. (2020) 100:3419–24. 10.3760/cma.j.cn112137-20200302-00557 33238672

[18] Khan AA Lodhi FS Rabbani U Ahmed Z Abrar S Arshad S. Impact of coronavirus disease (COVID-19) pandemic on psychological well-being of the Pakistani general population. Front Psychiatry. (2021) 12:564364. 10.3389/fpsyt.2020.564364 33510654 PMC7835389

[19] Elhadi M Alsoufi A Msherghi A Alshareea E Ashini A Nagib T. Psychological health, sleep quality, behavior, and internet use among people during the COVID-19 pandemic: a cross-sectional study. Front Psychiatry. (2021) 12:632496. 10.3389/fpsyt.2021.632496 33868049 PMC8044819

[20] Zavlis O Butter S Bennett K Hartman TK Hyland P Mason L. How does the COVID-19 pandemic impact on population mental health? A network analysis of COVID influences on depression, anxiety and traumatic stress in the UK population. Psychol Med. (2021) 16:1–9. 10.31234/osf.io/8xtdr 33722329 PMC8010290

[21] Liu C Liu D Huang N Fu M Ahmed JF Zhang Y. The combined impact of gender and age on post-traumatic stress symptoms, depression, and insomnia during COVID-19 outbreak in China. Front Public Health. (2021) 21:620023. 10.3389/fpubh.2020.620023 33553099 PMC7859527

[22] Pan KY Kok AAL Eikelenboom M Horsfall M Jörg F Luteijn RA. The mental health impact of the COVID-19 pandemic on people with and without depressive, anxiety, or obsessive-compulsive disorders: a longitudinal study of three Dutch case-control cohorts. Lancet Psychiatry. (2021) 8:121–9. 10.1016/S2215-0366(20)30491-0 33306975 PMC7831806

[23] Casagrande M Favieri F Tambelli R Forte G. The enemy who sealed the world: effects quarantine due to the COVID-19 on sleep quality, anxiety, and psychological distress in the Italian population. Sleep Med. (2020) 75:12–20. 10.1016/j.sleep.2020.05.011 32853913 PMC7215153

[24] Lovibond SH Lovibond PF. Manual for the Depression Anxiety Stress Scales, 2 Edn. Sydney: Psychology Foundation (1995).

[25] Bener A Morgul E Atak M Barişik CC. The impact of COVID-19 pandemic disease exposed with mental health in Turkey. Int J Clin Psych Mental Health. (2020) 8:16–9. 10.12970/2310-8231.2020.08.04

[26] Taheri M Esmaeili A Irandoust K Mirmoezzi M Souissi A Laher I. Mental health, eating habits and physical activity levels of elite Iranian athletes during the COVID-19 pandemic. Sci Sports. (2023) 38:527–33. 10.1016/j.scispo.2023.01.002 37362084 PMC10243596

[27] WHOQOL-BREF. Development of the World Health Organization WHOQOL-BREF quality of life assessment. WHOQOL Group Psychol. Med. (1998) 28:551–8. 10.1017/S0033291798006667 9626712

[28] Kroenke K Spitzer RL Williams JB. The PHQ-15: validity of a new measure for evaluating the severity of somatic symptoms. Psychosom Med. (2022) 64:258–66. 10.1097/00006842-200203000-00008 11914441

[29] Löwe B Decker O Müller S Brähler E Schellberg D Herzog W. Validation and standardization of the generalized anxiety disorder screener (GAD-7) in the general population. Med Care. (2008) 46:266–74. 10.1097/MLR.0b013e318160d093 18388841

[30] Buysse DJ Reynolds CF Monk TH Berman SR Kupfer DJ. The Pittsburgh Sleep Quality Index: a new instrument for psychiatric practice and research. Psychiatry Res. (1989) 28:193–213. 10.1016/0165-1781(89)90047-4 2748771

[31] Michielsen HJ De Vries J Van Heck GL. Psychometric qualities of a brief self-rated fatigue measure: the fatigue assessment scale. J Psychosom Res. (2003) 54:345–52. 10.1016/S0022-3999(02)00392-6 12670612

[32] Brailovskaia J Cosci F Mansueto G Miragall M Herrero R Baños RM. The association between depression symptoms, psychological burden caused by COVID-19 and physical activity: an investigation in Germany, Italy, Russia, and Spain. Psychiatry Res. (2021) 295:113596. 10.1016/j.psychres.2020.113596 33261924 PMC7688416

[33] Verma S Mishra A. Depression, anxiety, and stress and socio-demographic correlates among general Indian public during COVID-19. Int J Soc Psychiatry. (2020) 66:756–62. 10.1177/0020764020934508 32567466

[34] World Health Organization. Corona Virus Disease (COVID-2019) Situation Reports. (2023) Available online at: https://www.who.int/publications/m/item/weekly-epidemiological-update-on-covid-19-situation-reports (accessed March 30, 2023).

